# Assessing the Role of Cell-Surface Molecules in Central Synaptogenesis in the *Drosophila* Visual System

**DOI:** 10.1371/journal.pone.0083732

**Published:** 2013-12-26

**Authors:** Sandra Berger-Müller, Atsushi Sugie, Fumio Takahashi, Gaia Tavosanis, Satoko Hakeda-Suzuki, Takashi Suzuki

**Affiliations:** 1 Research Group Axon Guidance and Neuronal Connectivity, Max Planck Institute of Neurobiology, Martinsried, Germany; 2 CNRS 5273, Unité mixte de recherche STROMALab, Toulouse, France; 3 Dendrite Differentiation Group, German Center for Neurodegenerative Diseases (DZNE), Bonn, Germany; 4 Graduate School of Bioscience & Biotechnology, Tokyo Institute of Technology, Yokohama, Japan; Academia Sinica, Taiwan

## Abstract

A hallmark of the central nervous system is its spatial and functional organization in synaptic layers. During neuronal development, axons form transient contacts with potential post-synaptic elements and establish synapses with appropriate partners at specific layers. These processes are regulated by synaptic cell-adhesion molecules. In the *Drosophila* visual system, R7 and R8 photoreceptor subtypes target distinct layers and form *en passant* pre-synaptic terminals at stereotypic loci of the axonal shaft. A leucine-rich repeat transmembrane protein, Capricious (Caps), is known to be selectively expressed in R8 axons and their recipient layer, which led to the attractive hypothesis that Caps mediates R8 synaptic specificity by homophilic adhesion. Contradicting this assumption, our results indicate that Caps does not have a prominent role in synaptic-layer targeting and synapse formation in *Drosophila* photoreceptors, and that the specific recognition of the R8 target layer does not involve Caps homophilic axon-target interactions. We generated flies that express a tagged synaptic marker to evaluate the presence and localization of synapses in R7 and R8 photoreceptors. These genetic tools were used to assess how the synaptic profile is affected when axons are forced to target abnormal layers by expressing axon guidance molecules. When R7 axons were mistargeted to the R8-recipient layer, R7s either maintained an R7-like synaptic profile or acquired a similar profile to r8s depending on the overexpressed protein. When R7 axons were redirected to a more superficial medulla layer, the number of presynaptic terminals was reduced. These results indicate that cell-surface molecules are able to dictate synapse loci by changing the axon terminal identity in a partially cell-autonomous manner, but that presynapse formation at specific sites also requires complex interactions between pre- and post-synaptic elements.

## Introduction

During development, synaptic connections are established with extreme accuracy to build functional neuronal circuits. The formation of appropriate connections relies on distinct events. Initially, axons are directed to their target field, where they seek appropriate post-synaptic structures by forming transient contacts. When a suitable partner is recognized, these contacts become stable and synaptogenesis is initiated. Synaptic matchmaking is regulated by different families of synaptic adhesion molecules, including Cadherins, Ephrins/Eph, Neuroligins/Neurexins, SynCAMs, and LRRTM proteins (Leucine-rich repeat transmembrane)　[1–4].

The LRR adhesion molecule Capricious (Caps) is a crucial player of synaptic specificity at the neuromuscular junction in *Drosophila*. During the establishment of motorneuron innervation, Caps shows a specific and complementary expression pattern between a subset of muscles and the motorneurons that innervate them, and Caps accumulates at post-synaptic filopodia [5,6]. Furthermore, *caps* mutant motoraxons that normally express Caps abberantly target Caps-negative muscles, whereas expressing Caps in all muscles lead to the mistargeting of Caps-positive motoraxons to non-partner muscles [6]. These results suggest that Caps mediates neuromuscular recognition by homophilic axon-target binding. 

A similar mechanism has been suggested in the fly visual system. In the *Drosophila* eye, two photoreceptor subtypes (R7 and R8) innervate distinct synaptic layers in the medulla neuropile, M6 and M3, respectively. R7 and R8 axons utilize distinct sets of cell-surface molecules to recognize their specific synaptic layers: Caps, Golden goal (Gogo), Flamingo (Fmi), and Frazzled mediate M3 synaptic-layer targeting in R8s, whereas CadN and LAR are required for the recognition of the M6 layer by R7 axons [7–12]. Among these molecules, *caps* shows the most characteristic expression profile: *caps* is selectively expressed in R8 axons and in their recipient layer. In addition, the ectopic expression of Caps in R7 photoreceptors can redirect their axons to the R8-recipient layer [13]. Based on these results, it has been postulated that Caps promotes axon-target recognition by homophilic interaction. However, this assumption has not been tested to date.

Here, we show that *caps* mutant photoreceptors have a weaker phenotype in axon guidance than previously reported. We demonstrate that the recognition of the M3 layer by photoreceptors is not mediated by Caps homophilic axon-target interactions. Using a new genetic tool to visualize pre-synaptic sites, we found that Caps did not affect presynapse specification in R8 photoreceptors. Finally, we analyzed synapse formation in flies in which R7 axons were redirected to abnormal synaptic layers by overexpressing cell-surface molecules. When R7 axons were mistargeted to the M3 layer, R7s either maintained an R7-like synaptic profile or acquired a similar profile to R8s. When R7 axons were redirected to the M0-1 layer, the number of synapses was reduced. Altogether, we suggest that certain cell-surface molecules can transform the axon terminal identity, thereby changing their synaptic profile. Since photoreceptor axons are able to form synapses in some abnormal synaptic layers, but not all, we suggest that both pre- and post-synaptic partners participate in synapse formation. 

## Materials and Methods

### Fly strains and genetics

Flies were kept in standard *Drosophila* medium at 25 °C, except for Caps full length, Caps^ED^ and Caps^ID^ overexpression in R7 photoreceptors (29°C) and overexpression of *gogo* and *fmi* in R7 (20°C). The following lines were used: *ey3*.*5*FLP [14], FRT80B, *caps* ^c28fs^ [15], *caps* ^Δ1^ *trn* ^28.4^ [16], *Df*(*3L*)*Exel6118* [17], *Rh4-mCD8-4xGFP-3xmyc* (abbreviated as Rh4-GFP in the text), *Rh6-mCD8-4xGFP-3xmyc* (abbreviated as Rh6-GFP in the text) [12], 3Lcl FRT80 [18], *GMR-gal4*, *PM181-gal4* [10], *UAS-Caps-Ia4* [13,19], *UAS-caps* ^*ED*^ [19], *UAS-caps* ^*ID*^ [19], *Rh6-lexA::p65*, *Rh4-lexA::p65*, *lexAop2-brp-short* ^*cherry*^, *UAS-gogoT1* [12], *UAS-fmi* [20], *UAS-unc5* [21], and *UAS-caps* RNAi (Transformant ID: GD3046) [22]. The detailed genotypes are described in Table S1.

### Generation of transgenic flies

To generate the *Rh6-lexA::p65* and *Rh4-lexA::p65* lines, a 1731-bp fragment of the Rh6 promoter and a 2093-bp fragment of the Rh4 promoter were PCR-amplified using genomic DNA of *w* ^1118^ flies as a template. The primers CACC ACATGTTGCCTCATTGAATCAGAGAAAAATAGAAATTATCATCGC and TTCGAATGGCTGGTACTGGTGGCGCTT were used for the amplification of the Rh6 promoter, and CACCTCGCGTGTCATCCAGAACTTTG and CGGTCAACCCGATACCGAAC were used for Rh4. The 4 nucleotides CACC, which are required for directional cloning using the pENTR/D-TOPO® cloning kit (Invitrogen, USA) were added at the 5’ end of the forward primers. The fragments were cloned into the pENTR/D-TOPO vector. The inserts of Rh6 and Rh4 promoters were transferred to the destination vector *pBLexA::p65Uw* (Addgene, USA; [23]) using Gateway® LR Clonase™ enzyme mix kit (Invitrogen, USA). 

To generate the lexAop2-brp-short^cherry^ construct, a 717-bp fragment of GFP_S65T region was PCR-amplified from the genomic DNA of Bloomington 8484 flies. The XhoI and KpnI restriction sites were introduced by the forward primer GCGCCTCGAGGGTACCCAAAATGAGTAAAGGAGAAGAACTTTTCACTGGAGTTGTC and the XbaI restriction site by the reverse primer GCGCTCTAGATTATTTGTATAGTTCATCCATGCCATGTGTAATCCCAGC. The fragment was inserted into the pJFRC 18 *8XlexAop2-mCD8::GFP* vector (Addgene, USA; [22]) via XhoI and XbaI sites, and named pJFRC 18 *8XlexAop2-KpnI-GFP_S65T*. A 3043-bp fragment of *brp-short* ^*cherry*^ region was PCR amplified using the genomic DNA of *UAS-brp-short* ^*cherry*^ [24,25] as a template. The *brp-short* ^*cherry*^ includes the amino acids 473–1226 of the 1740 amino acid full-length BRP protein. The KpnI restriction site, the KOZAK (AATCAAA) site, and a start codon (ATG) were introduced by the forward PCR primer GCGCGGTACCAATCAAAATGGACTACAAGATCAAGCTGCGGGCCGCC and the XbaI restriction site was introduced by the reverse PCR primer GCGCTCTAGATTACTTGTACAGCTCGTCCATGCCGCCGGT. The fragment was inserted into the pJFRC 18 *8XlexAop2-KpnI-GFP_S65T* via KpnI and XbaI sites.

DNA for injection was prepared with Midiprep Kit (Qiagen, USA) and sent to BestGene Inc. (USA) for production of transgenic flies in the estimated attP40 landing site [26] for the *Rh6-lexA::p65* and *Rh4-lexA::p65*, and 28E7 landing site [27] for the *lexAop2-brp-short* ^*cherry*^.

### RT-PCR

Actin5C-Gal4 UAS-GFP flies (*yellow* gene was flipped out from Bloomington stock No. 4411) was crossed to UAS-*caps*RNAi (VDRC v3046). The flies were raised at 27°C and 10 adult flies for each genotypes were collected from Actin5C-Gal4 UAS-GFP/+; UAS-*caps*RNAi/+ and Actin5C-Gal4 UAS-GFP/+ control flies. Total RNA was purified using RNeasy mini (QIAGEN) and cDNA was synthesized using ReverTra Ace® qPCR RT Master Mix with gDNA Remover (TOYOBO). PCR was done by iPoof (Biorad). The primers used were: CapsF: CGGCAAAAAGTGTTAAATGCTGA, CapsR: AATTGACCATCAGGGTGTCGTC, GFPF: TTTTCAAAGATGACGGGAACTACAA, GFPR: GCTGTTACAAACTCAAGAAGGACCA. Caps primers were designed over 10kbp introns. The gel image was analyzed using the imageJ software.

### Immunohistochemistry and imaging

The experimental procedure for brain dissection, fixation and immunostaining was described previously [28]. We examined both sexes without distinction. The following primary antibodies were used: mAb24B10 (1:50 dilution, DSHB), rat antibody to CadN (Ex#8, 1:50, DSHB), rabbit antibody to GFP conjugated with Alexa488 (1:200, Molecular probes). For secondary antibodies, we used a series of Alexa488, Alexa568 and Alexa633 conjugated goat antibody to mouse, goat antibody to rabbit, goat antibody to rat (1:200, Invitrogen). 

Pictures were taken with Olympus confocal microscopes FV1000, Zeiss LSM780 and Nikon C2. Obtained images were processed with ImageJA 1.45b (NIH) and Photoshop CS5 (Adobe).

### Phenotype quantification

For synapse quantification, image stacks with a step size of Z=0.5µm were taken. 3D images were reconstituted and analysed with Imaris software (vs 7.4): After a set of R7 and R8 in the medulla was manually selected, Brp-short^cherry^ puncta were automatically identified by the spot detection module for each neuron (approx. synapse diameter was set to 0.35µm). Start- and endpoints of neurons were selected manually with the measurement points module. The most distal part of the R7 and R8 in the medulla was set as a start-point and the edge of CadN staining in the M5 layer as an end-point. Based on those data points, the distribution of Brp-short^cherry^ puncta along each neuron was calculated with a customized Imaris plugin written in Matlab (The Mathworks) as follows:  Each synapse coordinate was projected to a line segment representing the neuron from startpoint to endpoint. The line segment was divided into 10 bins and the number of synapses was calculated for each bin. Since R7 axons terminate deeper than the edge of CadN staining, additional bins with identical size where added for the quantification of the synaptic profile in R7 axons. Finally, the average number of Brp-short^cherry^ dots was calculated for each bin over all analyzed neurons. 

## Results

### Caps has a marginal role in R8 photoreceptor axon targeting

**Figure 1:**
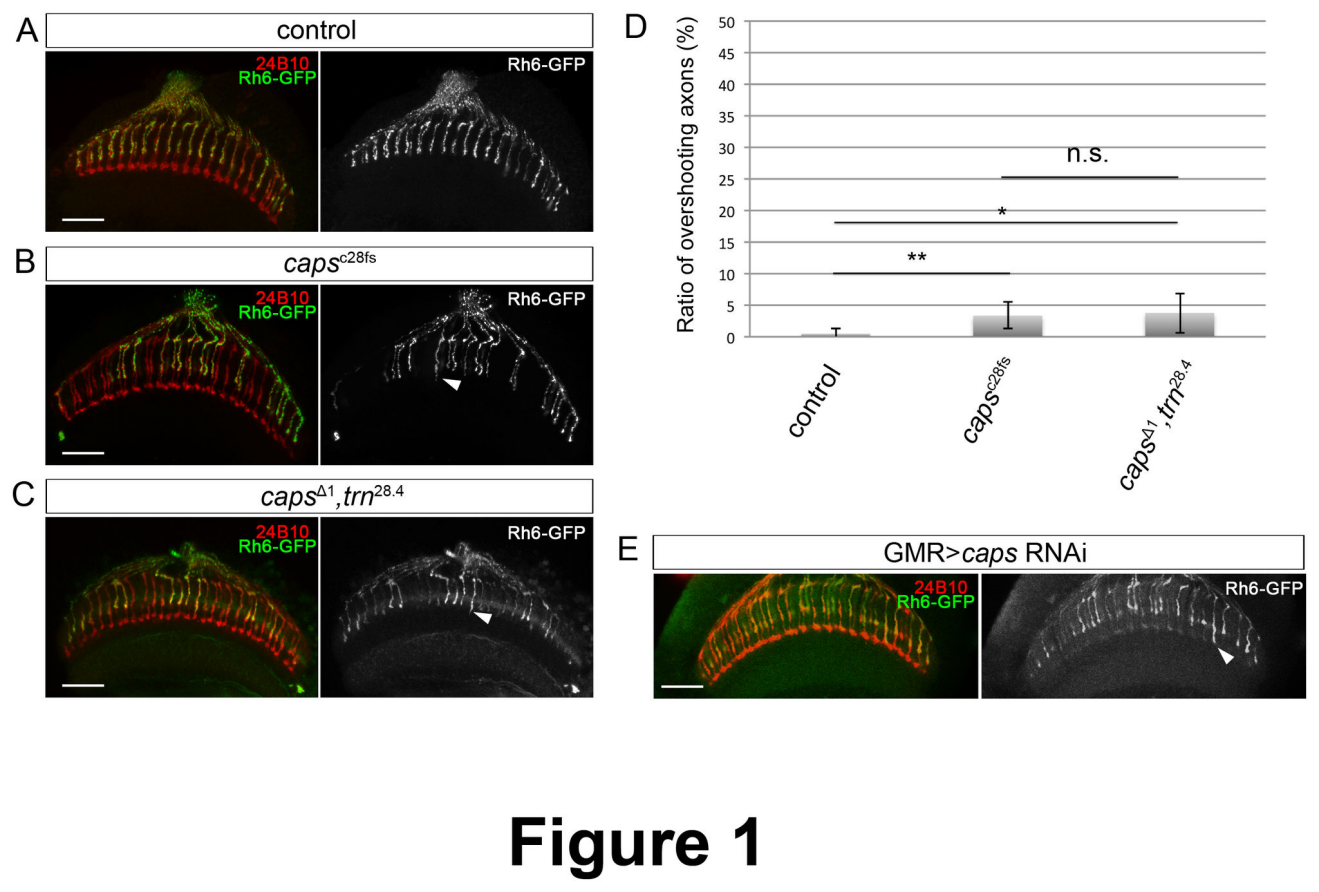
Caps has a marginal role in the guidance of R8 photoreceptor axons and is not redundant with Tartan. (A-C) Horizontal view of confocal sections of the medulla in flies carrying the Rh6-mCD8::GFP reporter to label R8 axons, stained with mAb24B10 (red) and anti-GFP (green). The GFP channel is shown on the right as black-and-white images. caps^c28fs^ eye-specific mosaic animals (B) and caps^Δ1^, *trn* ^28.4^ double mutant eye-specific mosaic animals (C) show overshooting axons (white arrowheads) that are a very mild phenotype compared to the wild-type (WT) control (A). (D) Quantification of the ratio of R8 axons that overshoot the M3 layer. * p<0.05, ** p<0.01 (t test). Error bars represent s.d. (E) caps RNAi induced a mild overshooting phenotype (white arrowhead). Scale bars represent 20 μm (A-C, E).

Using the EGUF/hid system to generate *caps* mutant mosaic eyes, it was shown that the targeting of *caps* ^*-/-*^ photoreceptor axons in the medulla is severely affected, and that R8 axons frequently fasciculate and undershoot or overshoot their proper target layer M3 [13]. To confirm this phenotype, we used the *ey3*.*5*FLP transgene, which drives FLPase expression specifically in the eye [14], to induce large clones homozygous for the *caps* null allele *caps* ^c28fs^ [15]. We found that R8 axons lacking Caps have a milder phenotype than previously described. We did not observe any axon bundling nor undershooting of the M3 target layer. However, *caps* ^*-/-*^ R8 axons extended over their target layer M3 and terminated at M4 or M5 at very low frequency (3.34±2.08%, p<0.01, Figure 1A, 1B and 1D). In contrast, *caps* mutant R7 axons did not show any targeting phenotype, as expected from the absence of Caps expression in R7 photoreceptors during pupal development [13]. The presence of the mutation (7-base pair deletion at the 28^th^ amino acid) was verified by sequencing (data not shown). To note, we observed a similarly subtle phenotype in R8 axon targeting when we expressed Caps RNAi in photoreceptors (2.75±1.97%, n=604, Figure 1E). RNAi efficiency was confirmed by RT-PCR (Figure S1).

The low penetrance of the *caps* loss-of-function phenotype in photoreceptor axons suggested that redundant proteins could ensure synaptic-layer targeting in the absence of Caps. Tartan (Trn) was a good candidate, since it is a close paralog of Caps [6] and has been reported to be redundant with Caps in boundary formation in the developing wing [29,30], leg segmentation [15], retinal epithelial integrity [16], dendritic targeting in the olfactory system [31], and axon targeting of motor neurons [5,32]. To check the redundancy between Caps and Trn in photoreceptor axon targeting, we analyzed the phenotype of *caps*/*trn* double mutant mosaic eyes. We used the combination of the *trn* null mutation *trn* ^28.4^ with a deletion that removes the entire Caps gene *caps* ^Δ1^ [16]. The double mutant phenotype was not enhanced compared to the *caps* single mutant (3.78±3.15%, p<0.68, Figure 1C and 1D), suggesting that the low penetrance of *caps* mutant phenotype is not due to a redundant function with *trn* in R8 axon targeting.

### Caps intra- and extra-cellular domain requirement for R7 mistargeting

**Figure 2:**
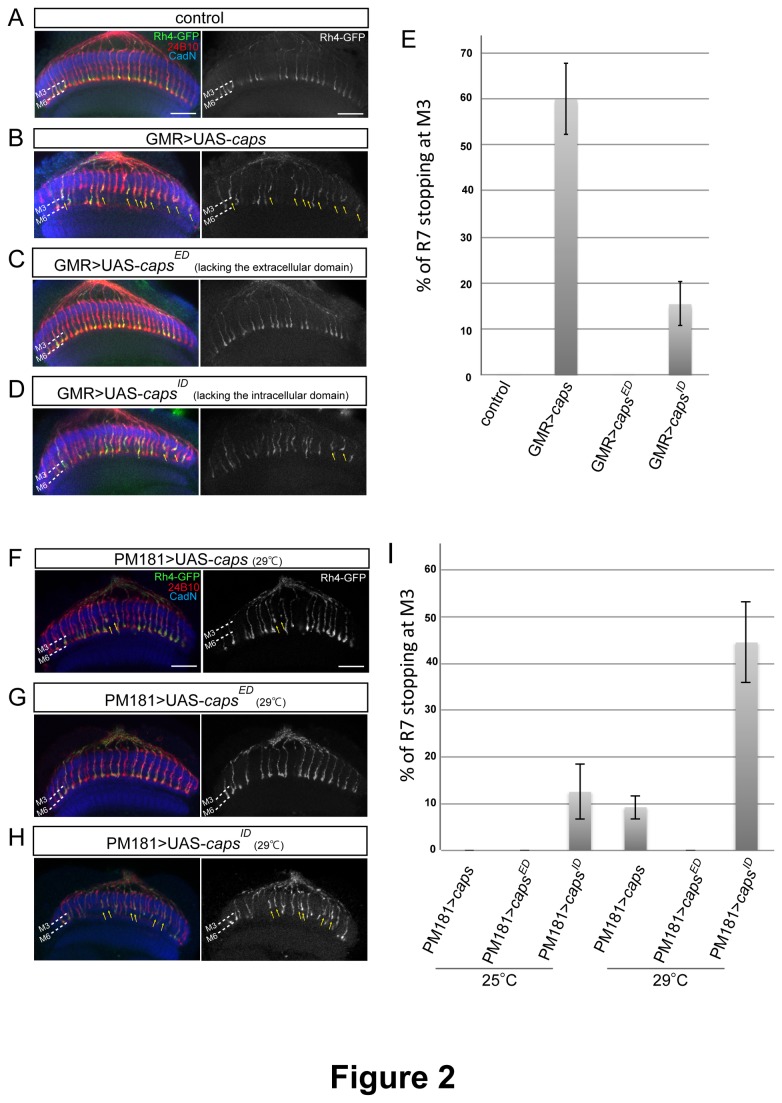
Overexpression of Caps truncations in photoreceptors. (A-D) Horizontal view of confocal sections of the medulla in flies overexpressing different fragments of Caps protein using the GMR-Gal4 driver. Photoreceptor axons are labelled with mAb24B10 (red), R7 photoreceptor axons with Rh4-GFP (green) and medulla layers with antibody to CadN (blue). The GFP channel is shown on the right as black-and-white images. Overexpressing Caps^ID^ (Deletion of the intracellular domain) redirects R7 axons to the M3 layer (yellow arrows in D), but to a lesser extent than full length Caps (yellow arrows in B). Overexpression of Caps^ED^ (deletion of the extracellular domain of Caps) does not result in a stopping phenotype (C). The wild-type (WT) control is shown in (A). (E) Quantification of the ratio of R7 axons that stop at the M3 layer. (F-I) Different fragments of Caps were overexpressed only in R7 using the PM181-Gal4 driver. When Caps^ED^ was expressed, no R7s stopped at M3 (G). Although both full length (F) and Caps^ID^ (H) generated R7 stopping at M3 (yellow arrows), Caps^ID^ induced a much stronger phenotype. (I) Quantification of the percentage of R7 axons that stopped at M3 at 25°C and at 29°C. Scale bars represent 20μm, error bars represent s.d.

Caps is a transmembrane protein that contains LRR motifs in its extracellular domain and a short intracellular domain. Although no known motif was identified in the intracellular part, the juxtamembrane region is highly similar in Caps and Tartan. Therefore, we wondered whether Caps acts as a simple adhesion molecule or as a receptor that mediates intracellular signalling via its cytoplasmic domain. It was previously shown using the GMR-Gal4 driver that Caps misexpression in both R7 and R8 photoreceptors induces R7 axon targeting to the M3 layer instead of their correct M6 layer (Figure 2A, 2B and 2E) [13]. We took advantage of the ability of Caps to retarget R7 axons to test the requirement of the intracellular and extracellular domains in axon targeting, by overexpressing deletion constructs in photoreceptor cells using the GMR-Gal4 driver. The ectopic expression of Caps^ED^ [19], a construct lacking the extracellular part, did not induce any mistargeting of R7 axons (Figure 2C and 2E), indicating that the ectodomain is essential for Caps function. Flies overexpressing Caps^ID^ [19], which lacks the intracellular domain, showed a much milder R7 stopping phenotype than Caps full length overexpression (15.4±4.7%, n=396, Figure 2D and 2E). As Caps can partially mediate axonal stopping without intracellular domain albeit much less effective, Caps extracellular domain seems to partly fulfil the function of the entire protein.

Since the GMR promoter drives gene expression in both R7 and R8 photoreceptors, it is possible that the observed R7 stopping phenotype is due to an increased adhesion between R7 and R8 axons, and not to axon-target interactions. It was previously shown that, using PM181-Gal4, Caps overexpression only in R7 can also generate an R7 stopping phenotype [13]. However, we found that the phenotype was much milder than when Caps was expressed in both R8s and R7s (0.00% at 25°C, n=460; 9.23±2.49% at 29°C, n=680; Figure 2F and 2I). This suggested that the stopping phenotype in Caps-overexpressing R7 axons relies at least partly on axon-axon homophilic interactions. To note, since Caps is endogenously expressed in R8s, we cannot exclude that the R7 stopping phenotype is due only to axon-axon homophilic interactions without contribution of axon-target interactions.

To further test the requirement of Caps domains for R7 axon stopping, we induced ectopic expression of Caps deletions specifically in R7 photoreceptors. Overexpressing Caps^ED^ did not show any phenotype again, which was consistent with the GMR-Gal4 experiment (Figure 2G and 2I). Surprisingly, overexpression of Caps without intracellular domain led to a much stronger phenotype than the full length protein (12.53±5.86% at 25°C, n=843; 44.47±8.61% at 29°C, n=761; Figure 2H and 2I). It appears that the requirement of the intracellular domain for R7 stopping is functionally opposite when Caps is overexpressed in both R7 and R8 or only in R7 axons. This indicates that the cytoplasmic part positively regulates axon-axon interactions, whereas it reduces Caps ability to adhere to the target layer. Altogether, these results indicate that Caps extracellular domain can mediate axonal recognition of the M3 layer, and that the cytoplasmic part regulates Caps activity. 

### Caps in the target layer does not guide photoreceptor axons

**Figure 3:**
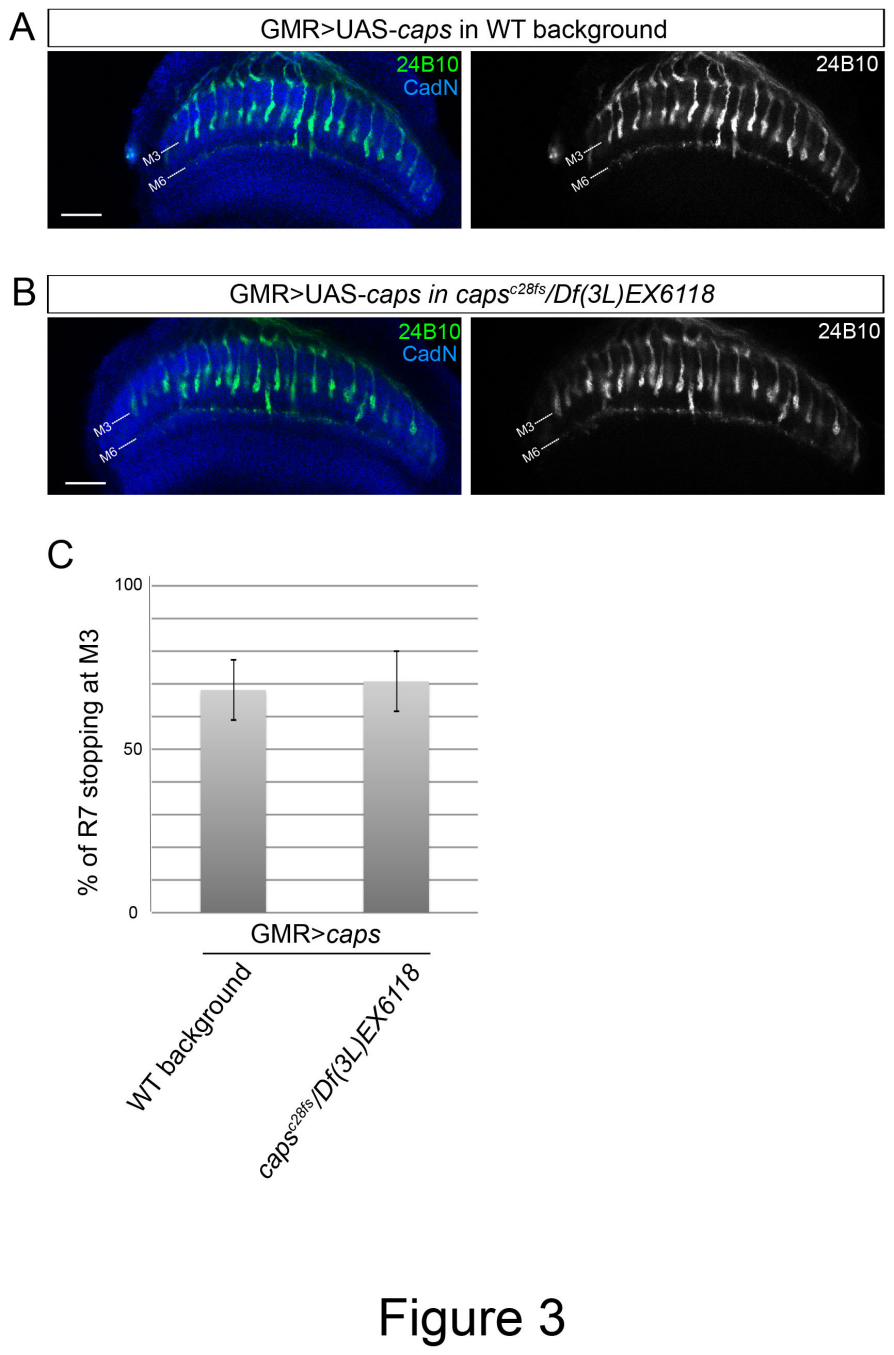
R7 mistargeting by Caps overexpression is not prevented by caps deletion from the target cells. (A, B) Caps was overexpressed in all photoreceptors by GMR-Gal4 in wild type (A) or in caps^c28fs^/*Df*(3L)*Exel6118* mutant background (B). Both R7 and R8 were labelled with mAb24B10 (green) and medulla layers with anti-CadN (blue). The 24B10 channel is shown on the right as black-and-white images. Deleting caps from the entire brain did not affect the amount of stopping R7 axons. Scale bars represent 20μm. (C) Quantification of the percentage of photoreceptors that stopped at M3. Control (68.11±9.32%, n=4 animals, 390 axons), caps mutant background (70.74±9.13%, n=7 animals, 491 axons), p>0.5 (t test).

During pupal stages, Caps is expressed specifically in R8 cells and in medulla layers comprising the R8-recipient layer, but not in R7 photoreceptors nor in the R7-recipient layer. In addition, R7 axons overexpressing Caps stop at the R8 target layer [13]. These results raised the possibility that Caps mediates axon targeting specificity by homophilic interactions between R8 axons and their target layer. To test this hypothesis, we ectopically expressed Caps in R7 (and R8) photoreceptors in transheterozygous flies (*caps* ^c28fs^/*Df*(*3L*)*Exel6118*). If Caps redirects R7 axons to the M3 layer by homophilic binding, we should observe a milder R7 stopping phenotype in the transheterozygous background since the target layer is mutant for *caps*. On the contrary, these animals displayed the same stopping phenotype as when the target layers are wild type (70.74±9.13% against 68.11±9.32% in control, p= 0.67, Figure 3A and 3B). This result indicates that Caps expressed in the target layer does not mediate M3 layer recognition. 

### Design and validation of genetic tools for synapse visualization

**Figure 4:**
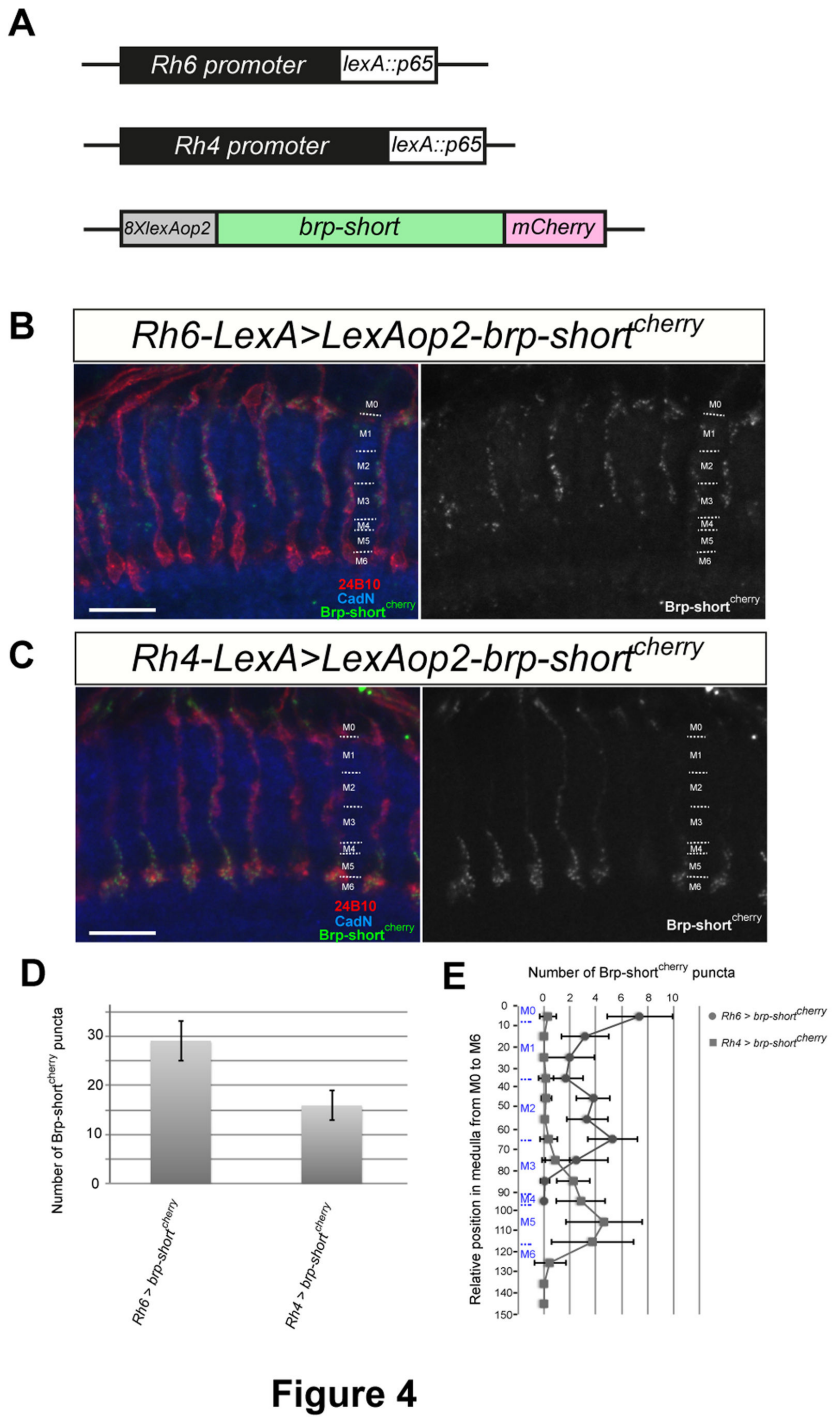
Genetic tools for the visualization of synapses in R7 and R8 photoreceptors. (A) Schematic of the genetic constructs allowing the expression of the pre-synaptic marker Brp-short^cherry^ in R7 or R8 phororeceptors. A 1731-bp fragment of the Rh6 promoter and a 2093-bp fragment of the Rh4 promoter were inserted upstream of lexA*::p65Uw*. The *Brp-short* ^*cherry*^ sequence was placed downstream of the 8x lexA operators.(B-C) Horizontal view of confocal sections showing the synaptic profile of R8s (B) and R7s (C). Brp-short^cherry^ fluorescence is shown in green, R7 and R8 photoreceptor axons were stained with mAb24B10 (red) and medulla layers by anti-CadN antibody (blue). The Brp-short^cherry^ channel is shown on the right as black-and-white images. Scale bars represent 10μm. (D) Quantification of the total number of Brp-short^cherry^ puncta per axons. (E) Distribution of Brp-short^cherry^ puncta of R7 (square) and R8 (circle) axons in the medulla layers. Error bars represent s.d.

We then wished to test the role of Caps in synapse formation. To visualize synapses, we generated constructs that allow the expression of a short version of Bruchpilot fused to the fluorescent protein mCherry (Brp-short^cherry^), known as a synaptic marker that localizes to the active zone at the neuromuscular junction [33] and in the calyx in the mushroom body [25]. The *Brp-short* ^*cherry*^ sequence was placed downstream of the *lexA* operator. The *rh6* and *rh4* promoters were fused to the *lexA* sequence to allow the expression of Brp-short^cherry^ in R8 and R7 photoreceptors, respectively (Figure 4A). 

When Brp-short^cherry^ was expressed under the control of the Rh6 promoter, we obtained a fluorescent signal appearing as dots along R8 axons in the medulla (Figure 4B). We first counted the number of synapses along R8 axon terminals, and found that there are about 30 synapses per R8 axon terminal in the medulla (Figure 4D). We then analysed the distribution of synaptic sites in the medulla neuropile. We subdivided the distal medulla from M0 to the edge of CadN staining at M5 in 10 identical layers, and counted the number of synapses in each layer. We found that R8 synapses were located all along the axonal shaft from the M0 to the M3 layer, but synaptic density was higher at the M0-1 and M3 layers (Figure 4E). These results are consistent with those obtained by EM analysis [34], indicating that the Brp-short^cherry^ protein properly localizes at active zones and can be used to study synapse formation.

We then looked at synapses in R7 axons using the Rh4 promoter (Figure 4C). Counting the Brp-short^cherry^ spots revealed that R7 photoreceptor axons contained less synapses than R8 axons (15.9±2.99, n=30, Figure 4D). Another striking difference between R7s and R8s was that synapses were concentrated at axon termini in R7 axons (Figure 4E). These observations are in line with what was previously observed by EM analysis [34]. Therefore, these flies constitute new powerful tools compatible with the UAS/Gal4 system to study synapses in a variety of genetic backgrounds. 

### Caps is not involved in synapse formation in R8 photoreceptors

**Figure 5:**
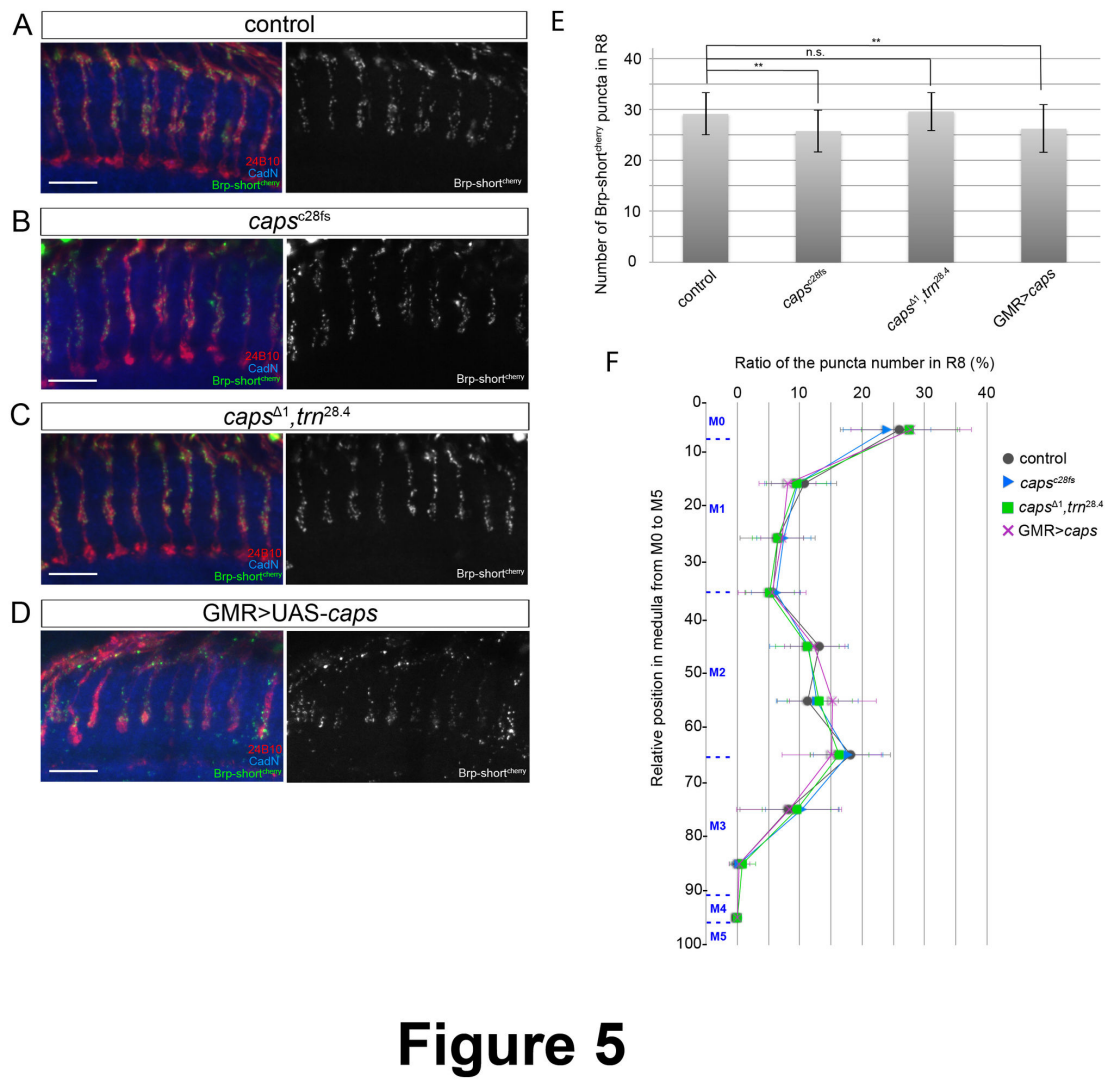
Caps is not involved in synaptogenesis. (A-D) Pre-synaptic active zones in R8 photoreceptor axons were visualized with Brp-short^cherry^ fluorescence (green). R7 and R8 photoreceptor axons were stained with mAb24B10 (red) and medulla layers by anti-CadN (blue). Wild type (A), caps^c28fs^ eye-specific mosaic mutant (B), caps^Δ1^, *trn* ^28.4^ eye-specific mosaic mutant (C), overexpression of Caps by GMR-Gal4 (D). Scale bars represent 10μm. (E) Quantification of the total number of Brp-short^cherry^ dots per R8 axon terminal for each genotype shown in A-D. **p<0.01 (t test). (F) Landscape of the distribution of Brp-short^cherry^ puncta along the axonal shaft of R8 photoreceptors in the medulla. Reducing or elevating caps expression levels does not affect R8 synaptic profile. Error bars represent s.d.

At the neuromuscular junction, it was shown that Caps accumulates at the tip of myopodia, and seems to stabilize contacts between pre- and post-synaptic partners [5]. However, it is not clear whether Caps serves only as a target recognition molecule, or if it is also a synapse organizer involved in early mechanisms of synapse formation at specific contacts. To test the role of Caps in synaptogenesis, we checked whether the synapse number and distribution is altered in *caps* mutant R8 axons. We expressed Brp-short^cherry^ in R8 photoreceptors of *caps* single mutant and *caps/trn* double mutant mosaic eyes and compared the number and the localization of synapses with control flies. In both mutants, we did not observe major changes in the average number of synapses per axons nor in their distribution in the medulla layers M0 to M3 (Figures 5A, 5B, 5C, 5E and 5F). We then tested the effect of Caps overexpression in photoreceptors using the GMR-Gal4 driver. Again, there was no significant difference in synaptic location profile in Caps-overexpressing R8 axons compared to the wild type, although there was a subtle decrease in total Brp-short^cherry^ number (Figure 5D and 5F). Therefore, both loss-of-function and overexpression experiments suggest that Caps does not have a major role in synapse formation at specific sites in R8 photoreceptor axons.

### Synaptic profile in misguided R7 photoreceptor axons

We next wondered how cellular interactions orchestrate synapse formation between defined combinations of neurons and at specific subcellular locations. In particular, we asked whether the recognition between pre- and post-synaptic partners is required for synapse formation. Thus, we decided to mistarget R7 photoreceptor axons to more superficial medulla layers than their usual target layer M6 in order to test if they are still able to form synapses at foreign synaptic layers. Since Caps misexpression in R7 photoreceptors can efficiently retarget R7 axons to the M3 layer, but Caps is not involved in synapse formation in R8 photoreceptors, we could use Caps overexpression in R7 axons to separate the processes of axon targeting and synaptogenesis, and to assess the influence of axon targeting on synapse formation at specific sites. We also overexpressed other cell-surface molecules to misguide R7 axons to different medulla layers and compared the outcome on synaptogenesis.

**Figure 6:**
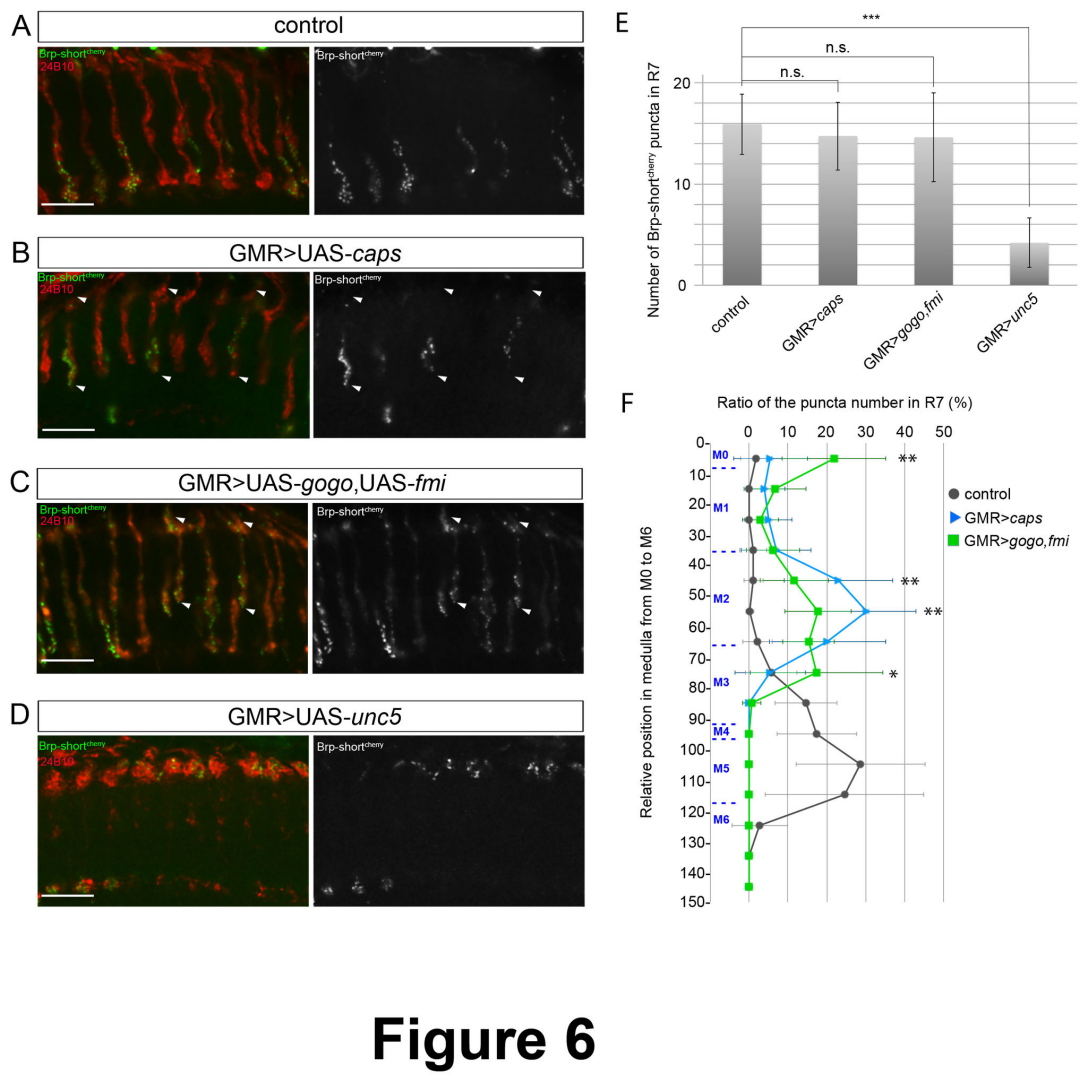
Pre-synaptic sites in R7 axons redirected to ectopic layers. (A-D) R7 and R8 axon terminals are stained with mAb24B10 (red), and presynaptic active zones in R7 axons are visualized by Brp-short^cherry^ fluorescence (green). R7 axons target the M6 layer in the wild type (A), whereas the R7 axons (marked by two white arrowheads) that overexpressed Caps (B) or Gogo and Fmi (C) were redirected to the M3 layer. Overexpression of Unc5 misdirected R7 to the more superficial layer M0-1 (D). Scale bars represent 10μm. (E) Quantification of Brp-short^cherry^ puncta per R7 axon terminal. The number of the puncta was reduced in Unc5-overexpressing R7s. (F) Brp-short^cherry^ distribution in R7 photoreceptor axons. In Caps-overexpressing R7s, the synaptic markers are essentially shifted from the M6 to the M3 layer. In contrast, Brp-short^cherry^ distribution in R7s overexpressing Gogo and Fmi was similar to that of R8 photoreceptors. * p<0.05, ** p<0.01, ***p<0.001 (t test) (GMR>caps VS GMR>gogo, *fmi*). Error bars represent s.d.

To test whether R7 axons can still form presynaptic terminals when they are redirected to an incorrect target layer, we overexpressed Caps in photoreceptors using the GMR-Gal4 driver. If synapse formation depended on both pre- and post-synaptic neurons, we would expect that R7 axons cannot form synaptic connections when they are misguided. Interestingly, we found that R7 axons mistargeted to M3 contained the same number of Brp-short^cherry^ as wild type R7 axons (Figure 6A, 6B and 6E). This suggested that misguided axons were able to form connections with inappropriate post-synaptic target. We then analyzed the localization of synapses in the different layers of the medulla as described above for R8 axons. We found that the main synaptic site of mistargeted R7 axons was at M3 layer, with only few synapses at the M1 and M2 layers (Figure 6B and 6F). Since this pattern of synaptic connections was different from R8 axons, which normally target the M3 layer and show synaptic sites at both M0-1 and M3 layers, we concluded that the localization of synapses does not only depend on axon targeting. Instead, both photoreceptor identity and axon targeting seems to define the location of synapse formation, and Caps-overexpressing R7 axons still maintain their R7 identity.

We then used alternative strategies to guide R7 photoreceptor axons to an incorrect target layer. The transmembrane proteins Golden Goal (Gogo) and Flamingo (Fmi) were shown to collaborate in R8 axons to target the M3 layer. When co-overexpressed in R7 photoreceptors, these proteins cause R7 axons to stop at the M3 layer [7]. We analyzed presynapse formation in R7 axons that overexpressed Gogo and Fmi using the GMR-Gal4 driver. Interestingly, we found that the synaptic profile was different from R7 axons which were misguided to the M3 layer by Caps overexpression: a large percentage of synapses were found at the M0-1 and M3 layers, with less synapses at M2 (Figure 6C and 6F). This synaptic profile was similar to R8 photoreceptor axons, indicating that Gogo and Fmi possibly instruct R7 axons to connect with R8 post-synaptic partners.

We then took advantage of the overexpression of Unc5, the repulsive Netrin receptor, in photoreceptors, which led to a complete stopping of R7 and R8 axons at the most superficial layer M0-1 (Figure 6D). Since the previous experiments showed that synapse number was not significantly affected in R7 axons misrouted to M3, we expected that R7 photoreceptor axons would be able to form synaptic connections also at the M0-1 layer. Interestingly, the number of pre-synaptic sites in R7 photoreceptors was greatly reduced when Unc5 was overexpressed (4.2±2.4 against 15.9±3.0 in wild type, p<0.001, Figure 6E). This result indicates that synapse number is not determined solely by the pre-synaptic neuron, but instead points towards a more communicative mechanism between pre- and post-synaptic partners to establish synaptic connections.

## Discussion

### A limited requirement of Caps in photoreceptor axon targeting and synapse formation

Our results suggest that the role of Caps in R8 photoreceptor targeting is less important than previously thought. Using the *ey3*.*5*FLP system, we did not obtain the strong bundling and overshooting phenotype that has been reported before in *caps* ^c28fs^ mutants [13,19]. However, we showed that *caps* mutant R8 axons have a very mild overshooting phenotype, indicating that Caps might still promote R8 axon stopping at their temporary target layer. To note, the phenotype was identical in mutants carrying the *caps* ^c28fs^ mutation or lacking the entire caps gene (*caps* ^Δ1^ in the *caps*/*trn* double mutant) or using Caps RNAi, further confirming that the observed phenotype is attributable to *caps* loss-of-function and that the penetrance is low. The strong *caps* phenotype reported before could be due to a sensitized background, since the transgenes used to generate *caps* mutant clones were different from ours. When we used the same fly stocks as in Shinza-Kameda et al., 2006, to generate MARCM clones, we also observed a bundling phenotype resembling what was previously reported (Figure S2). In Figure 1, however, we used a fly stock in which the 3^rd^ chromosome was recombined to add an FRT80 site. This recombination could have led to the removal of a background hit. 

Although the LRR protein Tartan was shown to act redundantly with Capricious in various contexts, we did not detect a significant increase in the R8 axon targeting phenotype in the double mutant. The low penetrance of the *caps* phenotype could be due to the functional redundancy with other proteins involved in R8 axon targeting to the M3 layer, including Gogo, Fmi, and Frazzled, or other LRR proteins.

### Caps intracellular domain may regulate Caps adhesive properties in photoreceptor axon targeting

In wing boundary formation and in synaptic specificity at the neuromuscular junction, the requirement of Caps intracellular domain seems to be context-dependent [19,29] suggesting that Caps can act as a mere adhesion molecule in some cases, but that the intracellular domain has a function in certain situations. To assess the role of Caps intracellular domain in photoreceptor axon targeting, we have performeda series of experiments comparing the contribution of the ectodomain and the cytoplasmic part in the guidance of axons to a specific synaptic layer.

We first observed that R7 axons could be retargeted to the M3 layer when Caps was overexpressed specifically in R7s, but the phenotype was much milder than when it was overexpressed also in R8s. These results indicate that the R7 stopping phenotype is at least partly due to Caps homophilic interactions between R7 and R8 axons. In contrast, when the Caps intracellular deletion (Caps^ID^) was overexpressed in both R7 and R8 photoreceptors, the R7 stopping phenotype was reduced compared to Caps full length. This suggests that Caps^ID^ has a reduced ability to induce axon-axon homophilic adhesion.

Interestingly, we found that Caps^ID^ induced a much stronger R7 stopping phenotype than the full length protein when overexpressed only in R7 axons. This indicates that Caps^ID^ can mediate stronger adhesion to the M3 layer than the entire protein. This adhesion probably involves a heterophilic binding partner at the M3 layer (see below). Strikingly, the R7 stopping phenotype was lower when Caps^ID^ was expressed in both R7s and R8s. This could be due the sequestration of a portion of the Caps ligand at M3 by CapsID overexpressed in R8 axon terminals. Thus, the Caps ligand at M3 would be partly saturated by Caps^ID^ present on the cell surface of R8s, letting less free binding site for Caps^ID^ expressed in R7s.

Overall, we propose a model in which the intracellular domain would regulate Caps adhesive properties, promoting homophilic binding and reducing adhesion to an unknown heterophilic partner at the M3 layer.

 To note, in the wild type situation, R8 photoreceptors express Caps, but R7s do not, thus R7-R8 homophilic interactions probably do not occur. In this scenario, only heterophilic axon-target interactions would take place in the endogenous situation. Since Caps full length seems to have a limited ability to promote axon-target adhesion, some regulating proteins, absent in R7 axons, may activate Caps in R8 photoreceptor terminals.

### A heterophilic ligand for Caps?

Due to the matching expression of Caps in axons and their corresponding targets, and to loss-of-function and gain-of-function data, it was suggested that synaptic specificity is encoded by Caps homophilic binding between pre- and post-synaptic elements both in motor neuron innervation and in photoreceptor targeting. However, this intuitive and attractive model was never assessed directly in these systems. We thus tested the requirement of Caps in post-synaptic targets. Since Caps overexpression in photoreceptors could misdirect R7 axons to the R8-recipient layer to the same extent when the target area was wild type or mutant for *caps*, Caps seems to recognize a heterophilic ligand at the M3 layer. However, we cannot exclude that the predominant Caps homophilic interactions between R7 and R8 axons mask the effect of axon-target interaction. 

 The hypothesis of a heterophilic ligand for Caps is in line with what was observed in the olfactory system. Caps expression in olfactory receptor neurons and in projection neurons does not match, and the dendrites of projection neurons target their correct glomeruli independently of their pre-synaptic partners, suggesting heterophilic interactions [31]. The use of a heterophilic ligand is further suggested by studies on boundary formation of the wing imaginal disc [30]. The identification of Caps ligand will be an important step to identify Caps-dependent mechanisms in synaptic specificity as well as in other contexts.

### Possible mechanisms controlling synaptic specificity by cell-surface molecules

We assessed the role of pre- and post-synaptic interactions in synapse formation by misdirecting photoreceptor axons to incorrect synaptic layers using the overexpression of cell-surface molecules. We showed that retargeting R7 photoreceptors to the M3 layer did not alter the number of pre-synaptic terminals, indicating that axons can form synaptic connections with non-partner neurons. Nevertheless, we cannot rule out that synapses of misguided axons are established with appropriate partners, even if these post-synaptic elements are not situated in the normal target layer: these ectopic synapses could be formed on the axonal shaft of the proper post-synaptic neurons going through the medulla column, and/or partner dendrites could be redirected to the layer where mistargeted axons stop. However, it seems unlikely that all the pre-synaptic sites of misguided axons are connected with original synaptic partners at a wrong layer.

The fact that mistargeted axons can establish synaptic contacts with novel partners is in line with several previous works [35–37]: photoreceptors axons that targeted an incorrect lamina cartridge, or an incorrect optic lobe, or coming from an ectopic eye were able to form presynaptic terminals with foreign targets. However, these reports indicate that the developmental program controlling presynaptic specification in photoreceptors is to a large extent cell-autonomous, or at least does not require specific post-synaptic partners. Our results suggest a more complicated mechanism for synaptogenesis involving communication between pre- and post-synaptic partners. Indeed, retargeting R7 photoreceptor axons to the medulla layer M0-1 leads to an important reduction of the synaptic marker number. This result argues against the idea that the total number of synapses per axon is fixed by the pre-synaptic cell. 

Our axon mistargeting experiments also shed light on the relationship between axon targeting and synaptogenesis. Since these two processes are intimately linked, it is difficult to assess how axon guidance can influence synapse formation. We showed in this paper that Caps does not have a role in synaptogenesis in photoreceptors, but Caps overexpression can be used to retarget R7 axons to an incorrect synaptic layer. Thus, Caps overexpression is a useful tool to assess the effect of axon guidance on synaptic specificity. When R7 axons were redirected to the R8-recipient layer by Caps overexpression, the pattern of synaptic connections was different from R8 axons. This result indicates that synaptic specificity does not depend solely on axon targeting. It also suggests that although Caps expression can confer the “R8 identity” to R7 axon terminals in terms of axon targeting, it does not have a role in determining the R8 synaptic profile on photoreceptor axon shaft. To note, the rhodopsin promoter Rh4, which was used to visualize synapses in mistargeted R7, was activated, suggesting that photoreceptor identity was unchanged. 

On the contrary, when we misguided R7 axons to the R8-recipient layer by Gogo and Fmi overexpression, the synaptic profile was similar to R8 photoreceptors. This indicates that, endogenously, Gogo and Fmi could control synapse formation at subcellular level in R8 photoreceptor axons, directly or indirectly. It also suggests that Gogo and Fmi co-expression can dictate photoreceptor neurons to transform their axonal identity to that of R8s.

A coordinated control of transcriptional factors, including Prospero and NF-YC, is known to control R7 cell identity, initially its axon targeting and later the rhodopsin expression during development [38]. Our data indicates that the synaptic profile is likely part (or downstream) of such cell fate determination, and that Gogo and Fmi can be important factors of the R8 genetic program. 

## Supporting Information

Figure S1 **Assessment of Caps RNAi efficiency by RT-PCR.** (A) Original gel image of RT-PCR. Lane 1,2: control RT-PCR against GFP mRNA. Lane 3,4: RT-PCR against caps mRNA. Lane 1,3: control flies without caps RNAi. Lane 2,4: flies with caps RNAi. caps RNAi was driven by Act5C-Gal4. (B) DNA quantification analysis using ImageJ. After background subtraction, the band intensity was calculated by cumulating brightness. The area of each gray mountain shape indicates the intensity of the band, thus the amount of DNA. The relative DNA amounts were indicated below each lane.
(PDF)

Figure S2 **Reproducing the caps mutant defects with original line with FRT2A.** *caps* ^c28fs^ MARCM clones were made by generating small *caps* mutant clones by *ey1x*-FLP.Exel stock. MARCM photoreceptor cells were labelled by GFP which is driven by Actin promoter Gal4. We see similar occasional defects that were reported previously, such as bundling or stalling R8 axons as indicated by arrows.
(TIF)

Table S1 **The detailed description of the genotypes used in the study.**
(PDF)
