# Supplementary figures and images for: Assessing the Role of Cell-Surface Molecules in Central Synaptogenesis in the Drosophila Visual System
Source: PLoS One. 2013 Dec 26;8(12):e83732. doi: 10.1371/journal.pone.0083732 (PMC3873376; doi:10.1371/journal.pone.0083732)

A

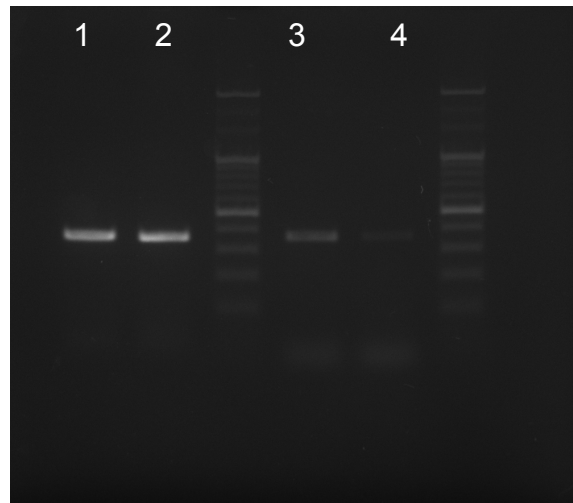

|                      |                       |     |   |  |             |   |
|----------------------|-----------------------|-----|---|--|-------------|---|
| <i>Actin5C</i> -Gal4 | UAS-GFP               | +   | + |  | +           | + |
|                      | UAS- <i>caps</i> RNAi | -   | + |  | -           | + |
| RT-PCR               |                       | GFP |   |  | <i>caps</i> |   |

B

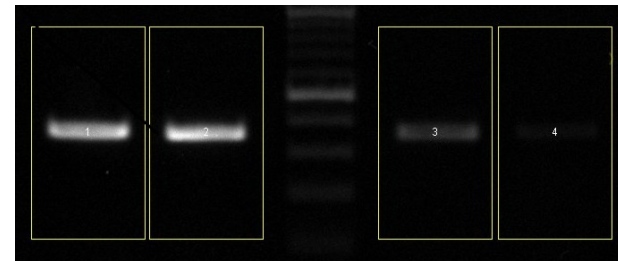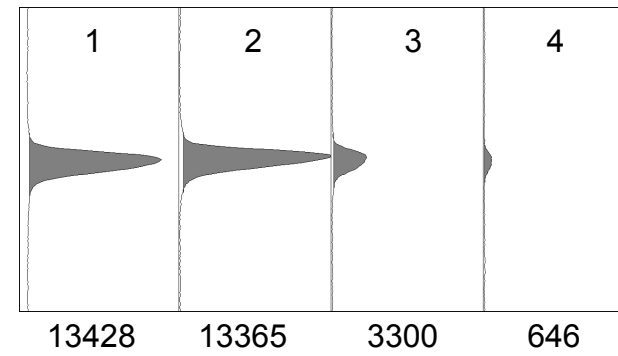

Supplement: Figure S1 — Assessment of Caps RNAi efficiency by RT-PCR. (A) Original gel image of RT-PCR. Lane 1,2: control RT-PCR against GFP mRNA. Lane 3,4: RT-PCR against caps mRNA. Lane 1,3: control flies without caps RNAi. Lane 2,4: flies with caps RNAi. caps RNAi was driven by Act5C-Gal4. (B) DNA quantification analysis using ImageJ. After background subtraction, the band intensity was calculated by cumulating brightness. The area of each gray mountain shape indicates the intensity of the band, thus the amount of DNA. The relative DNA amounts were indicated below each lane. (PDF) [file pone.0083732.s001.pdf]

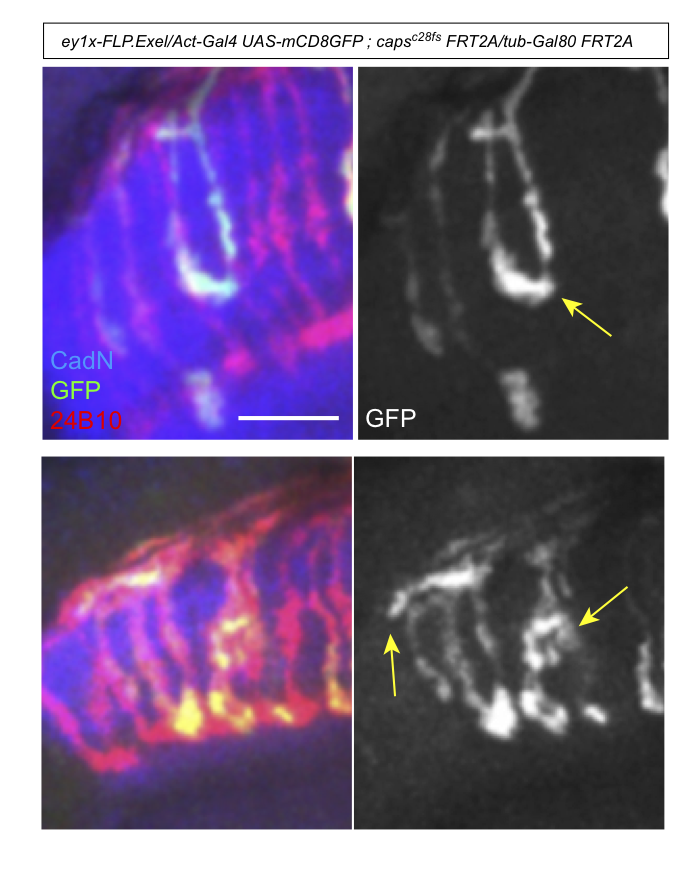

Supplement: Figure S2 — Reproducing the caps mutant defects with original line with FRT2A. caps c28fs MARCM clones were made by generating small caps mutant clones by ey1x-FLP.Exel stock. MARCM photoreceptor cells were labelled by GFP which is driven by Actin promoter Gal4. We see similar occasional defects that were reported previously, such as bundling or stalling R8 axons as indicated by arrows. (TIF) [file pone.0083732.s002.tif]
