# Supplementary material for: Assessing the Role of Cell-Surface Molecules in Central Synaptogenesis in the Drosophila Visual System
Source: PLoS One. 2013 Dec 26;8(12):e83732. doi: 10.1371/journal.pone.0083732 (PMC3873376; doi:10.1371/journal.pone.0083732)
Supplement: Table S1 — The detailed description of the genotypes used in the study. (PDF) [file pone.0083732.s003.pdf]

Table S1.

Figures	Genotypes
1A	*Rh6* -GFP, *ey* 3.5FLP(/+);; FRT80B/M(3)i^{55}, FRT80B
1B	*Rh6* -GFP, *ey* 3.5FLP(/+);; *caps*^{c28fs}, FRT80B/M(3)i^{55}, FRT80B
1C	*Rh6* -GFP, *ey* 3.5FLP(/+);; *caps*$^{\Delta 1}$, *trn*$^{28.4}$, FRT80B/M(3)i^{55}, FRT80B
1D	*Rh6* -GFP, *ey* 3.5FLP(/+);; FRT80B/M(3)i^{55}, FRT80B
1D	*Rh6* -GFP, *ey* 3.5FLP(/+);; *caps*^{c28fs}, FRT80B/M(3)i^{55}, FRT80B
1D	*Rh6* -GFP, *ey* 3.5FLP(/+);; *caps*$^{\Delta 1}$, *trn*$^{28.4}$, FRT80B/M(3)i^{55}, FRT80B
1E	GMR-Gal4/UAS-*caps* RNAi[GD3046]; *Rh6* -GFP/+
2A	*Rh4* -GFP(/+); GMR-Gal4/+
2B	*Rh4* -GFP(/+); GMR-Gal4/UAS-*caps-Ia4*
2C	*Rh4* -GFP(/+); GMR-Gal4/UAS-*caps*^{ED}
2D	*Rh4* -GFP(/+); GMR-Gal4/UAS-*caps*^{ID}
2E	*Rh4* -GFP(/+); GMR-Gal4/+
2E	*Rh4* -GFP(/+); GMR-Gal4/UAS-*caps-Ia4*
2E	*Rh4* -GFP(/+); GMR-Gal4/UAS-*caps*^{ED}
2E	*Rh4* -GFP(/+); GMR-Gal4/UAS-*caps*^{ID}
2F	*Rh4* -GFP/ UAS-*caps-Ia4* ; PM181-Gal4/+
2G	*Rh4* -GFP/ UAS-*caps*^{ED} ; PM181-Gal4/+
2H	*Rh4* -GFP/ UAS-*caps*^{ID} ; PM181-Gal4/+
2I	*Rh4* -GFP/ UAS-*caps-Ia4* ; PM181-Gal4/+
2I	*Rh4* -GFP/ UAS-*caps*^{ED} ; PM181-Gal4/+
2I	*Rh4* -GFP/ UAS-*caps*^{ID} ; PM181-Gal4/+
3A	GMR-Gal4, UAS-*caps-Ia4* /+
3B	GMR-Gal4, UAS-*caps-Ia4* /+; *caps*^{c28fs}, FRT80B/ *Df(3L)Exel6118*
3C	GMR-Gal4, UAS-*caps-Ia4* /+
3C	GMR-Gal4, UAS-*caps-Ia4* /+; *caps*^{c28fs}, FRT80B/ *Df(3L)Exel6118*
4B	*Rh6* -LexA::p65/ 8XLexAop2-*brp-short*^{cherry}
4C	*Rh4* -LexA::p65, 8XLexAop2-*brp-short*^{cherry} / +
4D	*ey* 3.5FLP(/+); *Rh6* -LexA::p65, 8XLexAop2-*brp-short*^{cherry} / + ; FRT80B/ M(3)i^{55}, FRT80B
4D	*Rh4* -LexA::p65, 8XLexAop2-*brp-short*^{cherry} / +
4E	*ey* 3.5FLP(/+); *Rh6* -LexA::p65, 8XLexAop2-*brp-short*^{cherry} / + ; FRT80B/ M(3)i^{55}, FRT80B
4E	*Rh4* -LexA::p65, 8XLexAop2-*brp-short*^{cherry} / +
5A	*ey* 3.5FLP(/+); *Rh6* -LexA::p65, 8XLexAop2-*brp-short*^{cherry} / + ; FRT80B/ M(3)i^{55}, FRT80B
5B	*ey* 3.5FLP(/+); *Rh6* -LexA::p65, 8XLexAop2-*brp-short*^{cherry} / + ; *caps*^{c28fs}, FRT80B/ M(3)i^{55}, FRT80B
5C	*ey* 3.5FLP(/+); *Rh6* -LexA::p65, 8XLexAop2-*brp-short*^{cherry} / +; *caps*$^{\Delta 1}$, *trn*$^{28.4}$, FRT80B/M(3)i^{55}, FRT80B
5D	GMR-Gal4, UAS-*caps-Ia4* / *Rh6* -LexA::p65, 8XLexAop2-*brp-short*^{cherry}
5E, F	*ey* 3.5FLP(/+); *Rh6* -LexA::p65, 8XLexAop2-*brp-short*^{cherry} / + ; FRT80B/ M(3)i^{55}, FRT80B
5E, F	*ey* 3.5FLP(/+); *Rh6* -LexA::p65, 8XLexAop2-*brp-short*^{cherry} / + ; *caps*^{c28fs}, FRT80B/ M(3)i^{55}, FRT80B
5E, F	*ey* 3.5FLP(/+); *Rh6* -LexA::p65, 8XLexAop2-*brp-short*^{cherry} / +; *caps*$^{\Delta 1}$, *trn*$^{28.4}$, FRT80B/M(3)i^{55}, FRT80B
5E, F	GMR-Gal4, UAS-*caps-Ia4* / *Rh6* -LexA::p65, 8XLexAop2-*brp-short*^{cherry}
6A	*Rh4* -LexA::p65, 8XLexAop2-*brp-short*^{cherry} / +
6B	GMR-Gal4, UAS-*caps-Ia4* / *Rh4* -LexA::p65, 8XLexAop2-*brp-short*^{cherry}
6C	GMR-Gal4, UAS-*gogoT1* / *Rh4* -LexA::p65, 8XLexAop2-*brp-short*^{cherry} / +; UAS-*fmi* /+
6D	GMR-Gal4, UAS-*unc5* / *Rh4* -LexA::p65, 8XLexAop2-*brp-short*^{cherry}
6E, F	*Rh4* -LexA::p65, 8XLexAop2-*brp-short*^{cherry} / +
6E, F	GMR-Gal4, UAS-*caps-Ia4* / *Rh4* -LexA::p65, 8XLexAop2-*brp-short*^{cherry}
6E, F	GMR-Gal4, UAS-*gogoT1* / *Rh4* -LexA::p65, 8XLexAop2-*brp-short*^{cherry} / +; UAS-*fmi* /+
S1	*w* ; Act5C-Gal4, UAS-*gfp* / UAS-*caps* RNAi[GD3046]
S2	*ey1x* FLP.Exel/ Act-Gal4, UAS-*mCD8GFP* ; *caps*^{c28fs}, FRT2A/ tub-Gal80, FRT2A
